# The Relationship between Negative Affect and Reported Cognitive Failures

**DOI:** 10.1155/2014/396195

**Published:** 2014-02-11

**Authors:** Tabitha W. Payne, Michael A. Schnapp

**Affiliations:** Department of Psychology & Neuroscience, Kenyon College, 118 Samuel Mather Hall, Gambier, OH 43022, USA

## Abstract

The purpose of this study was to expand our understanding of the range of negative affect associated with reported problems with everyday functions and activities, measured by the cognitive failures questionnaire (CFQ). Evidence from previous research indicates that individuals meeting criteria for mood disorders, such as major depression or seasonal affective disorder, experience cognitive deficits in memory and attention that can lead to problems with everyday activities reported in the CFQ. The Positive and Negative Affect Scale (PANAS) was used to assess potential correlations with a wider range of negative emotions. Findings for a sample of 129 college students revealed that negative affective experiences were significantly correlated with failures of memory and attention on the CFQ (*fear* = .41, *hostility* = .38, *sadness* = .28, and *guilt* = .43). Conversely, positive affect was negatively correlated with distractibility (*r* = −.21). Additional affective scales on the PANAS (e.g., *shyness and fatigue*) were also associated with higher reports of cognitive failures. The results provide converging evidence of a relationship between negative affective experiences and reported frequency of problems on the cognitive failures questionnaire.

## 1. Introduction

This study was conducted to further understand the relationship between the cognitive failures questionnaire (CFQ) and negative affect, such as emotions corresponding to depression and anxiety. Cognitive failures are defined as inabilities to successfully perform tasks that one might typically be able to do on a daily basis. Some examples include forgetting appointments, leaving mail unanswered for days, failure to notice street signs, and having to reread passages of text. Such failures in everyday activities are due to underlying problems in general cognitive functions related to distractibility and memory. In an attempt to measure such errors through the implementation of surveys, Broadbent et al. [1] developed the 25-item cognitive failures questionnaire. Although the scale was originally developed to measure a single general factor of reported frequency of listed cognitive difficulties, subsequent research using factor analysis has validated multiple subscales. Primary subscales include errors that can be characterized as having difficulty with attention/distractibility, memory, and motor function, also known as blunders [2]. Further research led to the addition of a fourth subscale for problems remembering names [3, 4]. Examples of cognitive failures for each of the factor are listed in Table 1.

Reported cognitive failures correlate with mood-related assessments for depression [5] and anxiety [2]. Interpretations of these findings can be dependent on whether CFQ scores are assessing traits. One view is that self-reports of high frequency cognitive failures could be indicative of a general ruminative cognitive style, which could increase vulnerability to negative affect, even depression [1]. Evidence for CFQ as a trait measure is supported by high retest correlations [1] and in findings of relationships with scales measuring neuroticism [1, 6]. Reported cognitive failures also correlate with trait anxiety [2, 7], again supportive of the notion that CFQ is measuring an enduring ruminative cognitive style that may be characterized as either neurotic worry or a tendency to complain about everyday slips in cognition [6].

Beyond this interpretation is the theory that the CFQ is comprised of errors in functionality that are actually markers of problems with basic cognitive processes, such as attentional focus. Evidence consistent with this perspective is findings that the CFQ correlates with performance on laboratory attention tasks, with higher reported cognitive failures associated with problems with sustained attention [8]. Additionally, Wallace et al. [9] found that several of the cognitive failure factors were correlated with adult symptoms of ADHD. As for validation of the memory factor in the CFQ, Groome and Grant [10] also found a significant inverse relationship between scores on the CFQ and retrieval-induced forgetting (*r* = −.432), which requires effectively tuning out irrelevant memory associates for selection of a target item in memory. Findings from these studies support the hypothesis that the CFQ correlates with objective measures of controlled attention and may be indicative of problems with basic cognitive processes. Additionally, these cognitive problems could be both subject to trait variation across individuals (based on nervous system tendencies—or high neuroticism) and also subject to intraindividual differences (reactions to negative events and trauma) that could be temporary in duration.

Problems with cognitive processing have been well documented with mood disorders. Most research has emphasized those with depressive disorders. Extreme negative affect associated with depressive disorders is linked to increased errors on assessments for a variety of executive functions, including verbal fluency [11, 12], attention [13], and working memory [14–16]. There have been a number of studies that have examined the relationship between depression and cognitive failures in various age groups. For instance, children who report symptoms of depression suffer from significantly more cognitive failures, such as overgeneralization errors, than nondepressed children [17]. On the opposite end of the age spectrum, findings from a study of healthy adults (over 65 years of age) determined that subjects' scores on the CFQ were not related to any of the demographic data including age [18]. Scores were, however, significantly associated with the geriatric depression scale (GDS), an inventory used to assess the levels of depression in an elderly population. Additionally, in a study with 103 college students the Beck Depression Inventory (BDI) significantly correlated with the CFQ (*r* = .43), [19]. Sullivan and Payne [5] also found that college students meeting the criteria for seasonal or even nonseasonal depression had significantly higher reports of cognitive failures in comparison to nondepressed individuals. The correlation between the Beck Depression Inventory and the CFQ was .47. Aside from additional findings of a relationship between cognitive failures and depressive symptoms, CFQ scores were found to be positively correlated with state anxiety symptoms even when the influence of trait anxiety and neuroticism were statistically controlled for [2], although there is also a significant association with trait anxiety [7]. Results of these studies are consistent with hypothesis that ruminative disorders characterized by negative emotional experiences, such as depression and anxiety, tax attentional processes and lead to noticeable changes in the experience of failures in routine activities that are reported in CFQ.

It is possible that the experience of negative affect, in general, without qualification of depression as a disorder, may also be linked to reports of cognitive failures. A range of affective experiences can be observed with the Positive and Negative Affect Scale (PANAS-X), [20]. The scale consists of the higher subscales measuring positive and negative affect, with three factors that comprise basic positive emotions (joviality, self-assurance, and attentiveness) and four basic negative emotions (fear, hostility, guilt, and sadness), along with several other affective scales (shyness, fatigue, serenity, and surprise). Watson and Clark found that both depression and anxiety assessments were positively correlated with the overall negative affect scale on the PANAS-X. The positive affect subscale was also shown to be positively correlated with social interaction. Additionally, the fear subscale of the PANAS-X correlated with measures of state anxiety (.74 HSCL anxiety scale, .56 STAI anxiety scale), the sadness subscale with measures of depression (.59 BDI, .69 depression subscale from the Hopkins symptoms checklist, .75 CES-D), and hostility scale with other measures of hostility (.55 SCL-90, .45 STAS). These findings from Watson and Clark support the validity of the PANAS-X, making it an appropriate measure not only to examine negative and positive affect in general, but also to assess more specific affective subscales that may be related to reported cognitive difficulties.

It is important to understand the extent to which the reported problems cognitive functioning measured by the CFQ relates to emotion and affective experiences, and the PANAS-X provides a valid measure of a range of negative emotions that could be linked to mood disorders. Given that the CFQ is related to depressive disorders, it is reasonable to assume that the experience of sadness will be associated with impairments; however, fear, guilt, and hostility may also be linked to cognitive failures. Any emotion or affective state that is characteristic of a ruminative mental state (such as in depression or anxiety) is likely to be correlated with cognitive problems. Additionally, positive affect either should not be related to the CFQ or should show a negative relationship. This study was designed to explore the range of affect subscales on the PANAS-X and the specific types of cognitive failures (i.e., problems with attention, general memory errors, blunders, and memory difficulty for names) on the CFQ to gain understanding of the magnitude of the relationship between both mood and cognitive functioning.

## 2. Method

### 2.1. Participants

The sample consisted of 129 participants (45 males and 84 females) who were current students or had just recently graduated from several small colleges in the central Ohio region (mean age = 22.78 years). Participants were recruited with campus flyers or through internet posts on an online research participation website for those enrolled in psychology courses and either were given course credit for volunteering or were compensated monetarily. Interested participants arranged individual appointments for survey completion.

### 2.2. Materials

#### 2.2.1. Cognitive Failures Questionnaire

This scale was used to assess how frequently participants commit errors in the completion of everyday tasks [1]. The cognitive failures included in the scale are 4 general factors of attentional difficulties, problems with memory, general blunders, and problems remembering names. One example of a cognitive failure relating to working memory would be forgetting why you just walked into a room. For each described cognitive failure, participants were asked to rate on a 5-point scale (0 = never, 4 = very often) how often they have experienced each of 25 given. Scores were compiled by adding total ratings, with scores range from 0 to 100. This assessment has been shown to be reliable in previous research, in terms of internal consistency (Cronbach's alpha was found to be .91), and also with test-retest reliability of .82 for over a 2-month period [21]. For the purpose of this study, reported ratings of the frequency cognitive failures were to be considered over a period of 2 weeks, as opposed to the standard 6-month appraisal in order to assess more current issues with cognitive impairment and more closely congruent with the PANAS time frame. This study used factors validated by Wallace [22] since the sample size (*N* = 709) was the largest of any study assessing the CFQ, while also having the highest comparative fit index (CFI = .96) and lowest expected cross-validation index [22].

#### 2.2.2. Positive and Negative Affect Scale—X

This measure consists of a list of 60 unique adjectives that describe feelings and emotions, like “cheerful,” “excited,” or “distressed,” in order to assess self-reported affect in the past week [20]. Participants are asked to rate the extent to which these emotions were felt over the past week on a 5-point scale (1 = very slightly/not at all, 5 = extremely). The scales examined consisted of positive affect (joviality, self-assurance, and attentiveness), negative affect (fear, hostility, guilt, and sadness), and additional scales (shyness, fatigue, serenity, and surprise), some of which are specifically states.

## 3. Results

### 3.1. Descriptives

#### 3.1.1. Positive and Negative Affect Scale

For the PANAS-X, factors were created by adopting ones that were validated with previous research [20] and adding up item ratings for each.  Table 2 lists the descriptive data for the PANAS factors. For the 10-item factor “negative affect,” the mean total score was 20.78 out of 50. For the 10-item factor “positive affect,” the mean total score was 28.80. Watson and Clark [20] also validated 11 subscales, the first four of which were basic negative emotion scales (the six-itemed “fear,” “hostility,” “guilt,” and five-itemed “sadness”). The next three subscales were comprised of the positive affect factors, (eight itemed “joviality,” six-itemed “self-assurance,” and six-itemed “attentiveness”). The final four subscales were comprised of 4 types of affective states that would not fall under a general positive of negative category (the four itemed “shyness” and “fatigue,” and three itemed “serenity” and “surprise”). The PANAS-X and affective factor scales demonstrated adequate internal consistency with our sample. The alpha reliability scores were .692 for general negative affect, and. 709 for general positive affect. The alpha reliabilities were equally high across the factor subscales, ranging from .751 for serenity to .698 for fear. Correlations between factors are listed in Table 4.

#### 3.1.2. Gender and Affect

Similar to the CFQ, we failed to find gender differences for most of the affective factor scales with the PANAS-X, with the exception of general positive affect, self-assurance, and serenity. For general positive affect, (*t*(122) = 2.21, *P* < .05) males (*M* = 30.57, SD = 6.05, SE = .91) reported slightly higher positive affect than females (*M* = 27.83, SD = 6.89, SE = .77). For self-assurance (*t*(124) = 2.39, *P* < .05) males (*M* = 17.47, SD = 4.54, SE = .69) reported higher levels in comparison to females (*M* = 15.31, SD = 4.93, SE = .54). Finally, for serenity (*t*(124) = 2.38, *P* < .05) males (*M* = 9.20, SD = 2.53, SE = .38) reported higher levels in comparison with females (*M* = 8.02, SD = 2.72, SE = .30).

#### 3.1.3. Cognitive Failures Questionnaire

The mean total score for the CFQ was 42.49 (SD = 12.34, SE = 1.09, range = 13–78). Refer to Table 2 for general descriptive statistics, including minimum and maximum points earned, total possible, and Cronbach's alpha. CFQ factors for this study were created by adding up the items validated by Wallace [22] for each of the four factors to create a total score for each factor grouping (memory errors, distractibility, blunders, and problems with names). The mean total score for the “memory” factor, comprised of 8 items, was 10.73 out of 32. The mean total score for “blunders” comprised of 9 items was 17.66 out of 36. The mean total score for the fourth factor, “names,” composed of only 2 scale items, was 4.12 out of 8. The CFQ as a whole, exhibited very high internal consistency (Cronbach's alpha = .89). No gender differences were found on either total CFQ scores, (*t*(127) = −1.104, *P* > .05) or any of the factor scales (*P* > .05). Among the individual factors, most had high internal consistency with *memory* errors yielding the highest value (.82); however, problems with *names* had fairly low reliability. All of the factor total scores in the CFG correlated significantly with one another, as listed in Table 3 correlation matrix. The names factor correlated the least with all other factors, whereas the remaining cognitive failures correlated more strongly.

### 3.2. Affect and Cognitive Failures

#### 3.2.1. Negative Affect and Cognitive Failures

The primary hypothesis was that negative affect would be correlated with cognitive failures, unlike experiencing positive affect. Correlational analysis indicated a direct and significant relationship between reported ratings for negative mood and scores on the CFQ, (*r* = .49, *P* < .001). Also consistent with this hypothesis is the finding that positive affect was not significantly correlated with overall total score on the CFQ (*r* = −.12, *P* > .05). Negative affect was significantly correlated with each type of cognitive failure, with memory, distractibility, and blunders yielding the highest correlations, (see Table 5 for a list of correlations between PANAS and CFQ scores). All of the negative affect factors (sadness, fear, guilt, and hostility) were correlated with failures of memory, distractibility, and blunders, but not problems with names.

#### 3.2.2. Positive Affect and Cognitive Failures

Additional analyses were performed to assess potential relationships between specific types of positive affect, (joviality, self-assurance, and attentiveness) and cognitive failures. Neither joviality nor self-assurance was found to be significantly correlated with cognitive failures; however, attentiveness was found to be significantly correlated with only one aspect of cognitive failures, distractibility. The relationship between attentiveness and distractibility was negative in the sense that higher levels of reported attentiveness were associated with lower levels of cognitive failures. Please see Table 5 for specific statistics.

#### 3.2.3. Other Affective States and Cognitive Failures

To investigate the relationship between other mood variables on the PANAS-X that fall outside the general description of positive and negative affect and cognitive failures, we conducted further analyses which revealed additional correlations. Fatigue and shyness correlated positively; however, serenity correlated negatively with three types of cognitive failures (memory, distractibility, and blunders). Surprise was the only variable correlated exclusively with difficulty with names.

#### 3.2.4. Participant Diagnoses and Reported Stress

Participants volunteered personal information on the demographic questionnaire regarding current mood disorder diagnoses and engagement in treatment. Only 8 participants disclosed having a current diagnosis and were in treatment. There were no statistical differences between these individuals and those not reporting such conditions on the PANAS or the CFQ. Individuals also reported whether or not they were aware of current events that were stressful. Fifty-eight individuals reported recent stressful events, while 68 did not report recent stressful life events. Results of an independent *t*-test indicated that reported stressful life events were not predictive of cognitive failures, (*t*(124) = −.63, *P* > .05). However, reports of stressful occurrences were associated with higher ratings for the extent of experienced negative affect (*t*(119) = −2.00, *P* = .048), equal variances not assumed), with individuals reporting stressful events having higher negative affect (*M* = 22.18, SE = 1.10), in comparison with those not reporting stressful events (*M* = 19.61, SE = .74).

## 4. Discussion

Findings from this correlational study expand our understanding of the affective spectrum and reveal that some specific moods are intricately related to reported problems with everyday slips in cognitive processing. The hypothesis that the reported intensity of experienced negative affect would be correlated with reported problems in everyday cognitive functioning was confirmed. Furthermore, each basic negative emotional factor (fear, sadness, guilt, and hostility) was significantly correlated with both scores on the whole CFQ, and each CFQ factor with the exception of having difficulty with names (see Table 5). It was expected that sadness would predict cognitive failures since we know that depression is linked to cognitive failures [5], and since the sadness factor on the PANAS-X has been shown to correlate with depression inventories [20]. There were no significant relationships with scores on the CFQ other than overall positive affect and attentiveness being negatively correlated with distractibility.

Of the four “other” affective factor scales, (shyness, fatigue, serenity, and surprise), only fatigue has been demonstrated by previous research to be correlated with higher frequencies of reported cognitive failures. Wallace et al. [9] found, with an undergraduate population using the Epworth sleepiness scale, that daytime sleepiness is a significant predictor of higher scores on the CFQ for three out of four factors: memory (*r* = .38), distractibility (*r* = .33), and blunders (.45); the study also showed comparable, but slightly lower, correlations with a population of military personnel. Results of this study confirm that higher experienced cognitive failures are related to not only fatigue, but also shyness. Since previous research has shown that shyness is correlated with worrying behavior [23], this affective factor can also be characterized by ruminations, which may in turn lead to cognitive failures. Surprise was the only negative affect factor that was associated with problems remembering names. Conversely, serenity was associated with fewer reported cognitive impairments.

The limitation of this research is that there are multiple explanations of the findings of correlations between CFQ scores and negative affect. One is that the CFQ is measuring a trait related to complaining—or an aspect of neuroticism, which may also overlap with the experience of negative affect. The other explanation is that intraindividual changes in cognition may occur when attentional resources are challenged by distracting thoughts related to stress and trauma. This explanation is consistent with research finding that depressed individuals show impaired working memory, as well as cognitive failures. Future research should examine the CFQ with repeated clinical samples to better understand how to identify whether the CFQ relates to actual temporary impairment.

These findings also help validate use of the cognitive failures questionnaire in understanding the range of emotions and mood disorders. This assessment inquires about everyday activities and correlates with human performance measures of controlled attention. The severity of reported cognitive failures is a good indication of negative affect and possibly a mental health conditions, such as depression or ADHD. Frequent reports of cognitive failures can be dangerous, and thus the CFQ is a recommended tool for identifying individuals in such vulnerable states.

## Figures and Tables

**Table 1:** Example items from the cognitive failures questionnaire.

Memory	Distractibility	Blunders	Names
Do you find you forget appointments?	Do you fail to notice signposts on the road?	Do you drop things?	Do you find you forget people's names?
Do you forget where you put something like a newspaper or a book?	Do you start doing one thing at home and get distracted into doing something else (unintentionally)?	Do you bump into people?	Do you fail to listen to people's names when you are meeting them?

**Table 2:** Descriptive statistics for total scores on PANAS-X and CFQ factors.

	Total	M	SD	Min	Max	Skew	Kurt	*α*
PANAS-X								
Positive affect	50.00	28.80	6.71	12.00	45.00	−.022	−.34	.89
Negative affect	50.00	20.78	7.09	10.00	44.00	1.15	1.07	.86
Fear	30.00	11.61	4.43	6.00	27.00	1.23	1.78	.83
Hostility	30.00	11.93	4.50	6.00	26.00	1.12	.76	.82
Guilt	30.00	11.44	4.89	6.00	28.00	1.15	.93	.85
Sadness	25.00	10.35	4.60	5.00	23.00	.92	.08	.87
Joviality	40.00	24.03	6.17	10.00	40.00	.16	−.14	.92
Self-assurance	30.00	16.05	4.89	7.00	29.00	.43	−.26	.82
Attentiveness	20.00	12.97	3.15	4.00	20.00	.00	−.36	.77
Shyness	20.00	6.95	2.67	4.00	15.00	.86	.16	.74
Fatigue	20.00	12.59	3.71	4.00	20.00	−.15	−.61	.82
Serenity	15.00	8.44	2.71	3.00	14.00	−.12	−.66	.77
Surprise	15.00	6.14	2.36	3.00	12.00	.53	.43	.69
Cognitive failures								
CFQ total	100.00	42.49	12.34	13.00	78.00	.607	.283	.89
Memory	32.00	10.73	4.63	1.00	26.00	.873	1.095	.82
Distractibility	36.00	17.66	4.82	6.00	29.00	.072	−.531	.75
Blunders	28.00	11.36	3.91	3.00	24.00	.708	.534	.70
Names	8.00	4.11	2.01	0.00	8.00	.219	−.809	.36

**Table 3:** Correlations between cognitive failure factors.

Names	Memory	Distractibility	Blunders
Memory	—		
Distractibility	.76**	—	
Blunders	.68**	.71**	—
Names	.26**	.29**	.32**
—			

(**P < .01).

**Table 4:** Correlation matrix of PANAS-X factors.

PANAS-X fACTORS												
	PA	NA	1	2	3	4	5	6	7	8	9	10
Positive affect												
Negative affect	−.06											
(1) Fear^N^	−.01	.85**										
(2) Hostility^N^	−.04	.79**	.53**									
(3) Guilt^N^	−.06	.82**	.61**	.62**								
(4) Sadness^N^	−.22*	.68**	.47**	.55**	.63**							
(5) Joviality^P^	.80**	−.22*	−.10	−.18*	−.25**	−.31**						
(6) Self-assurance^P^	.80**	−.09	−.05	.01	−.15	−.21*	.64**					
(7) Attentiveness^P^	.86**	−.17	−.12	−.09	−.11	−.24*	.60**	.64**				
(8) Shyness^O^	−.11	.41**	.39**	.25**	.47**	.28*	−.09	−.24**	−.16			
(9) Fatigue^O^	−.14	.52**	.35**	.46**	.47**	.41**	−.14	−.21*	−.15	.14		
(10) Serenity^O^	.46**	−.44**	−.32**	−.42**	−.34**	−.35**	.61**	.34**	.36**	.00	−.34**	
(11) Surprise^O^	.51**	.12	.26**	.11	.10	−.06	.50**	.48**	.31**	.16	−.03	.16

Note: N: negative affective factors, P: positive affective factors, O: other types of affective states.

(*P < .05, **P < .01).

**Table 5:** Correlations between affect and cognitive failures.

	Total CFQ	Memory	Distractibility	Blunders	Names
Positive affect	**−.12**	−.09	−.21*	−.08	.06
Negative affect	.49**	.42**	.41**	.50**	.19*
(1) Fear	.41**	.38**	.36**	.39**	.15
(2) Hostility	.38**	.31**	.27**	.42**	.17
(3) Guilt	.43**	.33**	.36**	.46**	.17
(4) Sadness	.28**	.26**	.27**	.24**	.07
(5) Joviality	**−.15**	−.11	−.17	−.16	.00
(6) Self-assurance	**−.09**	−.08	−.16	−.05	.10
(7) Attentiveness	**−.15**	−.14	−.22*	−.12	.06
(8) Shyness	.36**	.34**	.34**	.30**	.14
(9) Fatigue	.37**	.28**	.35**	.35**	.15
(10) Serenity	−.27**	−.26**	−.28**	−.25**	.04
(11) Surprise	** .10**	.15	−.02	.094	.18*

(*P < .05; **P < .01).
